# Effect of Conductive Polymers PEDOT:PSS on Exciton Recombination and Conversion in Doped-Type BioLEDs

**DOI:** 10.3390/polym15153275

**Published:** 2023-08-02

**Authors:** Jiayi Song, Yunxia Guan, Cheng Wang, Wanjiao Li, Xi Bao, Lianbin Niu

**Affiliations:** College of Physics and Electronic Engineering, Chongqing Normal University, Chongqing 401331, China; 2021110511011@stu.cqnu.edu.cn (J.S.); 17805426505@163.com (C.W.); wmzly888@163.com (W.L.); baoxixi2727@outlook.com (X.B.)

**Keywords:** organic light emitting diode, exciplex, conductive polymers, energy transfer, luminance efficiency, hole injection layer

## Abstract

Although the effect of the conductive polymers PEDOT:PSS on the electroluminescence performance of doped-type organic light-emitting diodes (OLEDs) has been studied, the process of PEDOT:PSS regulation of exciton recombination region and concentration within the deoxyribonucleic acid (DNA)-based doped-type BioLEDs is still obscure. In this study, we fabricated Bio-devices with and without PEDOT:PSS using varying spin-coating speeds of PEDOT:PSS. The Alq_3_:Rubrene-based BioLEDs achieve higher luminance (44,010 cd/m^2^) and higher luminance efficiency (8.1 cd/A), which are increased by 186% and 478%, respectively, compared to the reference BioLEDs without PEDOT:PSS. Similarly, the maximum luminance and efficiency of blue TCTA:TPBi exciplex-type BioLEDs are increased by 224% and 464%. In particular, our findings reveal that with an increasing thickness of PEDOT:PSS, the region of exciton recombination shifts towards the interface between the emitting layer (EML) and the hole transport layer (HTL). Meanwhile, the concentration of singlet exciton (S_1,Rub_) and triplet exciton (T_1,Rub_) increases, and the triplet-triplet annihilation (TTA) process is enhanced, resulting in the enhanced luminescence and efficiency of the devices. Accordingly, we provide a possible idea for achieving high performance doped-type BioLEDs by adding conductive polymers PEDOT:PSS, and revealing the effect of exciton recombination and conversion in BioLEDs given different PEDOT:PSS thicknesses.

## 1. Introduction

The organic light-emitting diodes (OLEDs) hold wide application prospects due to their low cost, high brightness, lightweight, and excellent color rendering characteristics [1,2,3]. In particular, OLEDs with high luminance efficiency are very significant for the wide application of displays and lighting [4,5]. Therefore, the efficiency of OLEDs largely depends on the efficient exciton recombination of holes and electrons [6,7]. Normally, holes and electrons are conveyed in the emitting layer (EML), where holes and electrons form an exciton to degradation radiation and emit visible light. In other words, high luminance efficiency of OLEDs is associated with the appropriate choice of materials and thickness [8]. The incorporation of a hole injection layer (HIL) as a buffer layer between the anode and the hole transport layer (HTL) is an essential approach for modifying the surface of the anode. It facilitates the enhancement of power function matching between the anode and HTL, improves the hole injection capability in OLEDs, and promotes carrier balance within the device [9].

Poly(3,4-ethylenedioxythiophene):poly(styrenesulfon-ate) (PEDOT:PSS) aqueous solution can be spin-coated to form a film with good electrical conductivity, light transmission, flexibility, and thermal stability, and for matching the energy level of indium tin oxide (ITO), which is widely used as a HIL [10]. In 2007, Wang et al. conveyed that by compounding MWCNT with PEDOT:PSS aqueous solution and using PEDOT:PSS:MWCNT as a cavity injection layer, the brightness intensity of the OLED is effectively increased [11]. In 2012, Zhao et al. used a PEDOT:PSS/molybdenum trioxide (MoO_3_) composite HIL to raise the anode power function to six eV, which resulted in a substantial decrease in the injection barrier between the anode and the HTL [12]. In 2014, Wu et al. used high conductivity PEDOT:PSS to modify graphene anodes, reduce the surface roughness of graphene anodes as well as the square resistance, and successfully prepare green fluorescent OLED devices with the highest efficiency of 1.09 cd/A [13]. In 2023, the mechanism of gradual morphological changes in aqueous solutions of highly conductive PEDOT:PSS is reported by Lee, successfully explaining the electrical and structural modulations that occur after immersion tests at various pH conditions [14]. However, most of the above studies have been conducted based on fluorescent OLEDs, while deoxyribonucleic acid (DNA)-based BioLEDs are relatively uncommon (they use DNA butanol solution as the EBL and hole buffer layer, resulting in so-called BioLEDs). Meanwhile, most efficiency analyses focus on utilizing the charge balance in a device design. However, none of them have incorporated the exciton recombination region and the influence of organic magnetic-field effects (OMFEs), which are two crucial factors for enhancing the photoelectric performance of devices. These factors should be considered to further improve the overall efficiency of organic electronic devices [15].

In this work, we investigated the effect of conductive polymers PEDOT:PSS on yellow and blue doped-type BioLEDs. As we all know, the use of PEDOT:PSS as HIL was able to effectively improve the photoelectric performance of the Bio-device. The PEDOT:PSS-HIL BioLEDs all showed different degrees of improvement compared to the reference BioLEDs without PEDOT:PSS incorporation. By adding PEDOT:PSS HIL and hole buffer layers, the hole concentration in EML increases with the increasing thickness of PEDOT:PSS. Therefore, PEDOT:PSS HIL yellow 8-hydroxyquinoline aluminum (Alq_3_):5,6,11,12-tetraphenylnaphthacene (Rubrene) BioLEDs has a 478% and 186% higher maximum luminance efficiency and luminance than that without PEDOT:PSS BioLEDs. In addition, blue exciplex-type BioLEDs were prepared using 4,4′,4′′-tris (N-carbazolyl) triphenylamine (TCTA):2,2′,2′′-(1,3,5-benzinetriyl)-tris(1-phenyl-1-H-benzimidazole) (TPBi) as the EML. The maximum luminance and luminance efficiency of blue TCTA:TPBi exciplex-type BioLEDs were increased by 224% and 464%. Furthermore, we investigated the effect of PEDOT:PSS on the exciton recombination region of the device. As the thickness of PEDOT:PSS increased, the exciton recombination region moved toward the EML/HTL interface, allowing excitons to be better confined in the EML, thus, improving the luminance efficiency of the doped-type BioLEDs.

As we all know, organic magnetic-field effects (OMFEs) offer significant advantages for probing OLEDs micro-physical processes, which are directly observed by analyzing magneto-electroluminescence (MEL) responses [16,17,18]. Using the MEL non-contact detection technology, we found that as the thickness of PEDOT:PSS increases, the concentration of triplet exciton (T_1,Rub_) increases, and the triplet-triplet annihilation (TTA, $T_{1,\mathrm{Rub}}+T_{1,\mathrm{Rub}}\to S_{1,\mathrm{Rub}}+S_{0}$) [19,20,21] process is enhanced, resulting in the enhanced luminescence of the Bio-device. For example, at 400 µA, as the PEDOT:PSS thickness increases, the TTA intensity of the Bio-device is enhanced, while the luminescence of the Bio-device increases from 2885 cd/m^2^ to 4026 cd/m^2^. At the same time, the singlet exciton (S_1,Rub_) is generated through the Förster resonance energy transfer (FRET) process, which enhances the prompt fluorescence (PF) of the Bio-device, resulting in the enhanced luminescence of the Bio-device.

## 2. Materials and Methods

To explore the important influence of PEDOT:PSS, we fabricated four kinds of PEDOT:PSS-HIL Bio-devices [0, 1000, 2000, 3000 revolutions per minute (rpm)] named as devices A1, A2, A3, and A4. First of all, the structure of the four doped-type BioLEDs prepared in this paper is ITO/PEDOT:PSS (X rpm)/DNA (20 nm)/4,40-(cyclohexane-1,1-diyl) bis(N-phenyl-N-p-tolylaniline) (TAPC, 50 nm)/Alq_3_:Rubrene (10:1, 40 nm)/Alq_3_ (50 nm)/lithium fluoride (LiF, 1 nm)/Aluminum (Al, 120 nm). Where X equals 0 (corresponding to device A1, no spin-coated PEDOT:PSS), 1000 (device A2), 2000 (device A3), and 3000 (device A4). Here, using the organic small molecule material Alq_3_ and Rubrene as the main functional layer, PEDOT:PSS as the hole injection layer (HIL), and DNA as the electron blocking layer (EBL). Additionally, to investigate the effect of PEDOT:PSS on the luminance performance and electron-hole recombination region of exciplex doped-type BioLEDs, four exciplex-based devices with a structure of ITO/PEDOT:PSS (Y rpm)/DNA (20 nm)/TAPC (50 nm)/TCTA (5 nm)/TCTA:TPBi(1:1, 10 nm)/TPBi(45 nm)/LiF (1 nm)/Al(120 nm) were prepared. Where Y equals 0 (corresponding to device B1, no spin-coated PEDOT:PSS), 1000 (device B2), 2000 (device B3), and 3000 (device B4), to further verify the effect of PEDOT:PSS on the electron-hole recombination region of doped-type BioLEDs. The PEDOT:PSS, Alq_3_ Rubrene and TAPC were purchased from Xi’an Polymer Light Technology Corp in Xi’an, China. The TCTA and TPBi were purchased from Changchun Tuocai Corp in Changchun, China. The DNA was purchased from Sinopharm Chemical Reagent Corp in Shanghai, China.

Before vacuum evaporation, special lotion and deionized water were used to rinse the dirt out of the ITO glass. The ITO glass was then sonicated with deionized water, anhydrous ethanol, and acetone in turn for 15 min to improve the smoothness of the ITO’s appearance and the stability of the device, and the substrates were put into an oven and dried. PEDOT:PSS deionized water solution was spin-coated onto ITO glass at 1000, 2000, and 3000 rpm for 50 s and then baked in a vacuum glove box at 120 °C for 15 min. Devices A1 and B1 were not spin-coated with PEDOT:PSS deionized water solution. After annealing for 20 min, DNA butanol solution was spin-coated onto the PEDOT:PSS films at 2000 rpm for 40 s, followed by baking at 40 °C for 10 min.

Under the condition that the vacuum degree was better than 10^−4^ Pa, another functional layer was evaporated. Usually, the evaporation rate of organic materials is 0.02–0.06 nm·s^−1^, and the evaporation rate of Al electrodes is 0.15 nm·s^−1^. Each functional layer thickness and evaporation rate were measured with a film thickness tester (SI-TM206C, Changchun, China). The electroluminescence (EL) spectra of all devices were tested by a portable spectroradiometric luminance meter (PR-655, Changchun, China). For measuring the magnetic effect curve, the device was fixed to the cold head of the cryogenic system between the electromagnets (Lakeshore 643, Beijing, China). Loading bias voltage V and current I to the devices was achieved via Keithley 2400 multimeter. Throughout the testing process, all devices were at a constant voltage.

## 3. Results and Discussion

### 3.1. Photoelectric Performance of Yellow Doped-Type BioLEDs

**Figure 1:**
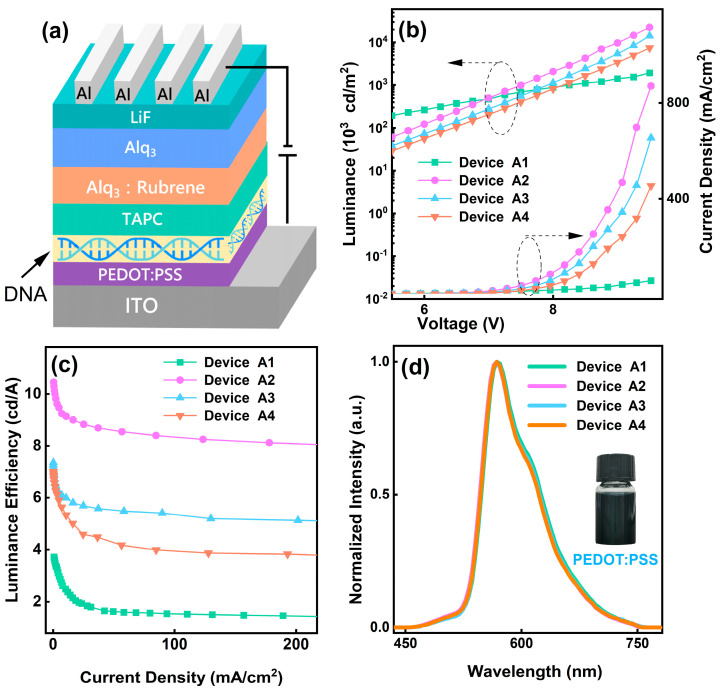
Bio-device structure, optical properties, EL spectra. (**a**) Bio-device structure used in this study. (**b**) J-L-V curve of devices A1–A4, (**c**) luminance efficiency-J curve of devices A1–A4, (**d**) Normalized electroluminescence (EL) spectra of 9 V.

**Figure 2:**
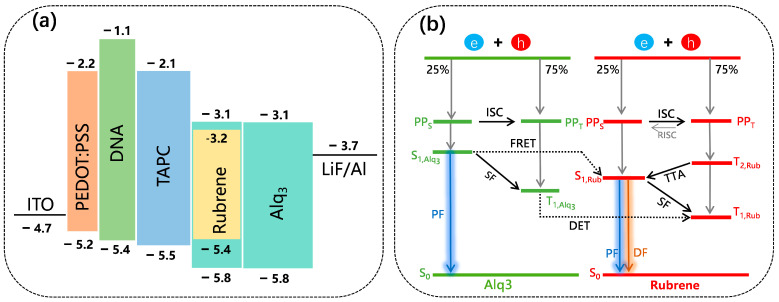
(**a**) The energy level structure of devices A1–A4. (**b**) The schematic of the EL processes in EML and microscopic process in devices A1–A4.

In order to investigate the electron transport mechanism, we fabricated four Bio-devices based on PEDOT:PSS. The device structure is illustrated in Figure 1a. Here, using organic small molecule material Alq_3_ doping Rubrene as the main functional layer, TAPC as HTL, PEDOT:PSS as HIL, and the buffer layer, DNA, as EBL. Device energy-level structures are shown in Figure 2a. As seen, the highest occupied molecular orbitals (HOMO) difference between Alq_3_ and TAPC is 0.3 eV, leading to the hole carriers injected from TAPC to Alq_3_ to be cushy [22]. Meanwhile, since the electron mobility of TAPC is quite poor (9.4 × 10^−6^ cm^2^/Vs) [23] and the lowest unoccupied molecular orbital (LUMO) offset between Alq_3_ and TAPC is about 1 eV, due to how the electron carriers injected from Alq_3_ to TAPC are difficult. In addition, the LUMO energy level of the guest material Rubrene is lower than the LUMO energy levels of the host materials Alq_3_, and the HOMO energy level is higher than the HOMO energy levels of the host materials Alq_3_, as such, carrier traps can be formed effectively in the doping layer. Therefore, the majority of holes and electrons will stack between TAPC and the electron transport layer (ETL), which promotes exciton formation [24].

Figure 1b shows J-L-V characteristics of Bio-devices with PEDOT:PSS HIL at different spin-coated speeds (1000 rpm, 2000 rpm, and 3000 rpm) and without the PEDOT:PSS device A1. At the same bias V, the current density J shows a monotonously decreasing relationship with the PEDOT:PSS spin speeds. Inside the Bio-device, electrons and holes form a compound current I in the organic layer, and the total current I of the Bio-device can be expressed as [25]

(1) $$I=I_{e}\prime +I_{h}=I_{h}\prime +I_{e}\;,$$

where $I_{e}\prime$ and $I_{h}\prime$ are leakage currents of electron and hole in the Bio-device, respectively, and I_e_ and I_h_ are currents I of the electron and hole in the Bio-device, respectively. Since all functional layers of Bio-devices A1–A4 are identical except for the HIL, the $I_{e}\prime$ and I_e_ of the Bio-device remain constant under the same electric field. The potential barrier of hole injection into the Alq_3_ layer is decreased, as adds PEDOT:PSS HIL and PEDOT:PSS spin-coated speeds decrease from 3000 rpm to 1000 rpm, which increases the hole currents, and causes the current of the Bio-device to raise. The trend of current density J can be explained by the equation [26] J = I/S, where S is the effective light-emitting area of the Bio-device (2 mm × 2 mm). As the PEDOT:PSS spin-coated speeds decrease, the overall Bio-device I increases, leading to the J to gradually increase, suggesting that a thicker PEDOT:PSS buffer layer [i.e., the highest occupied molecular orbitals (HOMO) of ITO (HOMO_ITO_ = −4.7) > the HOMO of PEDOT:PSS (HOMO_PEDOT:PSS_ = −5.2) > the HOMO of DNA (HOMO_DNA_ = −5.4)] can effectively improve the hole injection. For example, at 9 V, the J of devices A1, A2, A3, and A4 are about 28.1, 252.6, 201.4, and 127.8 mA/cm^2^, respectively.

As shown in Figure 1b, at the same bias V, the luminance increased with the decreased spin-coated speeds of the PEDOT:PSS. According to the analysis of the organic electroluminescent Bio-device mechanism, organic electroluminescence (EL) is a bimolecular (hole and electron) compound luminescence process. Therefore, under the action of the electric field, the luminescence of devices A1–A4 is a proportional function of the product of hole concentration and electron concentration. Under general conditions, the compound luminance intensity of the Bio-device can be expressed as [27]

(2) $$B=p\eta_{e}\eta_{h},$$

where, B represents the compound luminance intensity of the Bio-device; p represents the probability of radiation-induced jumps in the complex of the hole–electron pair; η_e_ and η_h_ are the electron concentration and hole concentration in Bio-device, respectively. From the above, it can be seen that PEDOT:PSS can significantly promote holes from PEDOT:PSS into Alq_3_. Therefore, at the same V, the hole concentration in the Alq_3_:Rubrene layer increases with the increasing thickness of PEDOT:PSS. Meanwhile, the LUMO difference between Alq_3_ and TAPC is about 1 eV, due to how the electron carriers injected from Alq_3_ to TAPC are difficult. Thus, the luminance of devices A1–A4 increases with the increase of PEDOT:PSS thickness at the same bias V. Similarly, compared to device A1 without PEDOT:PSS (15,400 cd/m^2^), device A2 (44,010 cd/m^2^), A3 (39,564 cd/m^2^), and A4 (34,180 cd/m^2^) with PEDOT:PSS have a significantly increased maximum luminance. PEDOT:PSS HIL doped-type BioLEDs have a 186% higher maximum luminance than comparable BioLEDs.

**Figure 3:**
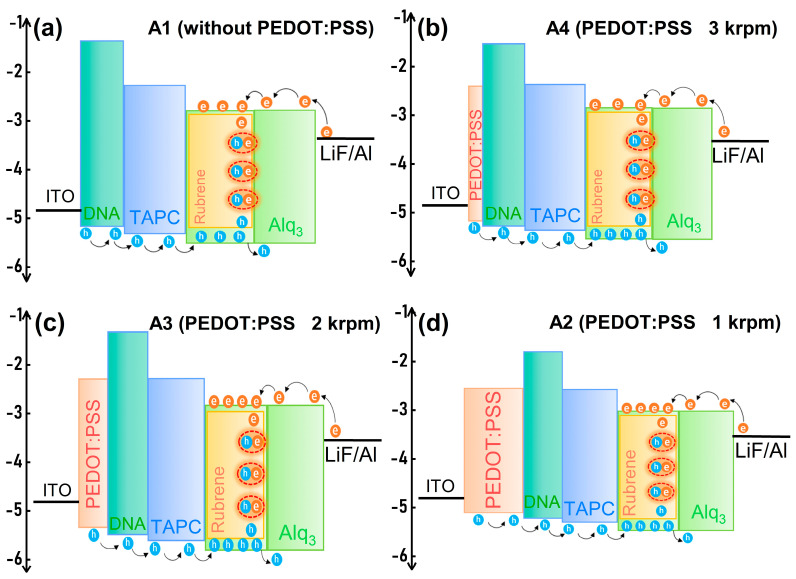
Schematic diagram of the exciton recombination region for devices A1–A4. (**a**) Device A1 without the PEDOT:PSS HIL. (**b**) Device A4 with the PEDOT:PSS HIL of 3000 rpm. (**c**) Device A3 with the PEDOT:PSS HIL of 2000 rpm. (**d**) Device A2 with the PEDOT:PSS HIL of 1000 rpm.

Luminance efficiency trends are consistent with the J and luminance. As shown in Figure 1c, it is clear that the luminance efficiency increases with an increased thickness of PEDOT:PSS, while maintaining the same current density (J). This suggests that a thicker PEDOT:PSS layer contributes to a higher luminance efficiency in the device. The addition of PEDOT:PSS increases the concentration of electrons and holes in the Alq_3_:Rubrene layer, improves the exciton recombination rate, and the efficiency of Bio-device is effectively improved. For example, at 200 mA/cm^2^, the luminance efficiency of device A1, A2, A3, and A4 are about 1.4 cd/A, 8.1 cd/A, 5.1 cd/A, and 3.8 cd/A, respectively. PEDOT:PSS HIL doped-type BioLEDs have 478% higher luminance efficiency than comparable BioLEDs at 200 mA/cm^2^. This shows that PEDOT:PSS is good for improving the luminance efficiency of the Bio-device. The linear spin-coated speeds’ dependence on the luminance efficiency can be analyzed by the working mechanism for devices A1–A4 shown in Figure 3. When the spin-coated speeds of PEDOT:PSS decreases, as depicted earlier, the I_h_ in devices A4–A2 increases and the I_e_ remains constant, leading to the hole–electron recombination region toward the EML/HTL interface, thus, confining the excitons well within the Alq_3_:Rubrene layer [28]. As a result of charge balance within the Alq_3_:Rubrene layer and the excellent exciton confinement, the 1000 rpm PEDOT:PSS HIL device A2 exhibits higher maximum luminance efficiency compared to without the PEDOT:PSS HIL device A1.

Figure 1d shows the EL spectra of devices A1–A4 and the PEDOT:PSS solution. As the PEDOT:PSS spin-coated speeds increase, the EL peaks of devices A1–A4 remain consistent, all at 568 nm and 608 nm. Rubrene solid-state thin film has two EL peaks at 568 nm and 608 nm, indicating that devices A1–A4 are all Rubrene emitting [29,30]. Notably, devices A1–A4 both show small EL peaks at 520 nm, indicating the presence of an exciton recombination region in Alq_3_. Furthermore, this observation suggests that the emissions of devices A1–A4 all originate from the exciton recombination region located between TAPC and the electron transport layer (ETL). This consistency supports the previous discussion regarding the importance of exciton recombination in this specific region for efficient light emission in these devices. In other words, by using PEDOT:PSS as the HIL, the charge balance enables efficient confinement of the exciton within the Alq_3_:Rubrene layer, and the region of exciton recombination is consistently confined to within the Alq_3_:Rubrene layer, although, there is a certain displacement of the recombination region.

### 3.2. Effect of Injection Current on the Bio-Device MEL Response

**Figure 4:**
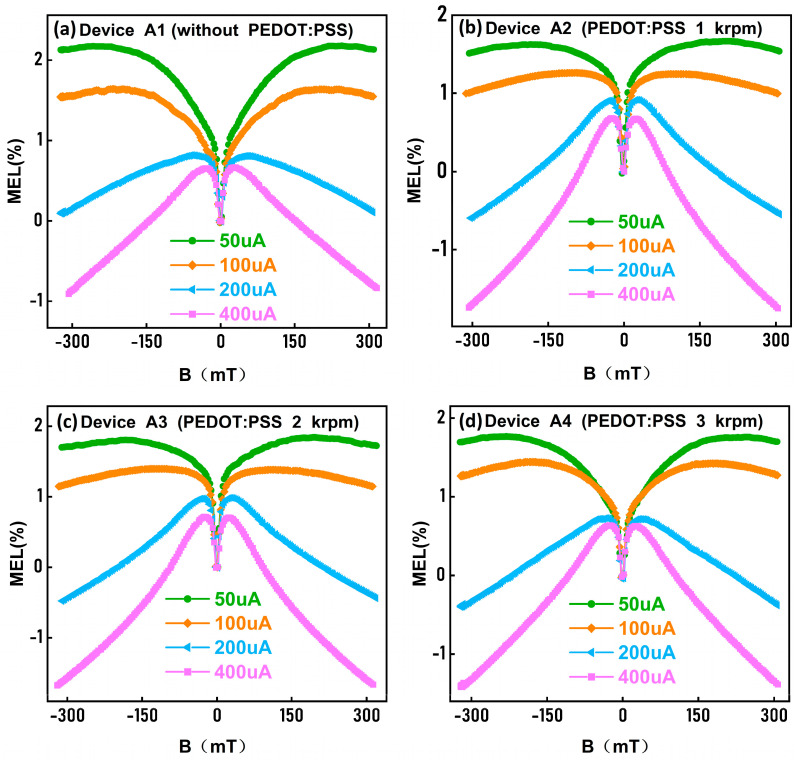
The MEL responses at different injection currents. (**a**) Device A1 without the PEDOT:PSS HIL. (**b**) Device A2 with the PEDOT:PSS HIL of 1000 rpm. (**c**) Device A3 with the PEDOT:PSS HIL of 2000 rpm. (**d**) Device A4 with the PEDOT:PSS HIL of 3000 rpm.

The evolution of the internal exciton spin mixing process in BioLEDs can be effectively probed in real-time by using organic magnetic field effects (OMFEs) in a contactless and damage-free manner. Therefore, to further verify the influence of the electron transport layer, MEL responses of different PEDOT:PSS thickness Bio-devices are also studied. The MEL Definition:

(3) MEL=ΔEL/EL×100%=[EL(B)−EL(0)]/EL(0)×100%,

where: EL(B) and EL(0) indicate the electroluminescence intensity of Bio-devices when a magnetic field (B) is present and when it is absent, respectively. Figure 4 shows the variation of MEL with the bias current I for devices A1, A2, A3, and A4.

As shown in Figure 4, the MEL responses of devices A1–A4 have a similar linear pattern. When B increases, the MEL responses increase rapidly in the |B| < 25 mT range, increase slowly (50 μA and 100 μA) and then decrease rapidly (200 μA and 400 μA) in the 25 mT < |B| < 300 mT range. Clearly, all of the low magnetic field range MEL responses originated from the ISC process [31,32,33]; this process is expressed as ${\mathrm{PP}}_{S}\to {\mathrm{PP}}_{T}$. The ISC process, SF process ($S_{1,\mathrm{Rub}}+S_{0}\to T_{1,\mathrm{Rub}}+T_{1,\mathrm{Rub}}$) [34,35], and TTA process ($T_{1,\mathrm{Rub}}+T_{1,\mathrm{Rub}}\to S_{1,\mathrm{Rub}}+S_{0}$) [19,20,21] are the dominant processes at a high magnetic field range in devices A1–A4. In addition, the MEL amplitude of devices A1–A4 decreases with the bias I increase, which belongs to the normal I dependence. Surprisingly, as the current increases, there is a shift from a positive to a negative value of MEL. This is because as the current increases, devices A1–A4 change from the SF process to the TTA process, which also belongs to the normal I dependence.

In general, due to Coulombic attraction, singlet polaron pairs (PP_s_) and triplet polaron pairs (PP_T_) form singlet and triplet excitons (S_1_ and T_1_, respectively) [36]. According to the spin statistic, the ratio of triplet state to singlet state is 1:3 [37,38], and the PP_S_ and the PP_T_ are regulated by HFI and the occurrence of ISC. Compared to PP_T_, PP_S_ is more ionic, and at a large bias I, PP_S_ is dissociated into free electrons and holes, which are shown in Figure 2b. Therefore, when the I in devices A1–A4 increases, the dissociation of PP_S_ in the Rubrene molecule is enhanced, resulting in a weaker ISC process between the PP_S_ and PP_T_. As shown in Figure 2b, when a larger bias I is injected, the concentration of T_1,Alq3_ and T_1,Rub_ in devices A1–A4 increases, which promotes the TTA process. At the same time, more PP_S_ are dissociated into free electrons and holes, and the concentration of S_1,Alq3_ and S_1,Rub_ in devices A1–A4 consequently decreases, suppressing the SF process. In other words, the SF process dominates when there is a low bias I, and as the bias I increases, the SF intensity weakens, leading to a shift from a positive to a negative value of MEL. At a high I, the TTA process dominates, and as the bias I increases, the TTA intensity increases. Therefore, as the bias I increases, MEL responses change from the SF to the TTA process, and the MEL values change from positive to negative.

The analysis of the electroluminescence performance of devices A1–A4 was conducted by magnetic field effects. From Figure 4, it is evident that the devices A1–A4 MEL are dominated by TTA processes, respectively, at 400 μA. As is well-reported, in the doped system, excitons can be formed by two processes: the (1) direct charge trapping (DCT) process, where electrons and holes in the EML are directly compounded to form excitons under the action of energy level traps; and (2) energy transfer process, where electrons and holes first form excitons in the Alq_3_ molecule, and then form excitons on the Rubrene molecule through a Förster resonance energy transfer (FRET) [39,40] and Dexter energy transfer (DET) [41] energy transfer processes. From the above, it can be seen that PEDOT:PSS can significantly promote holes from PEDOT:PSS into Alq_3_. Therefore, at the same I, the hole concentration in the Alq_3_:Rubrene layer increases with increasing thickness of PEDOT:PSS.

As shown in Figure 2b, the DET process increases the concentration of T_1,Rub_ and T_2,Rub_ in the Bio-device, resulting in more T_2,Rub_ being converted to S_1,Rub_ through the TTA process. Meanwhile, the FRET process increases the concentration of S_1,Rub_ in the Bio-device, which is advantageous for the luminance of the Bio-device. It is evident that the TTA process is enhanced simultaneously, further enhancing the luminance of the Bio-device when the thickness of PEDOT:PSS increases. For example, at 400 µA, the MEL values of the devices A1–A4 are −0.80%, −1.73%, −1.60%, and −1.36% at 300 mT, respectively. It shows that the intensity of the TTA process of the Bio-device increases simultaneously with the increase of the PEDOT:PSS thickness. Therefore, under the same bias I, as the PEDOT:PSS thickness increases, the hole concentration of devices A1–A4 increases, and it is also easier for the TTA process to occur, making the maximum luminance of the Bio-device enhanced. In Figure S1, when the bias I is 400 μA, the corresponding luminance of devices A1–A4 are 2885, 8022, 5959, and 4026 cd/m^2^, respectively. As the thickness of PEDOT:PSS increases, the maximum luminance of the Bio-device decreases. In other words, devices A1, A2, A3, and A4 reach the maximum luminance of 15,400, 44,010, 39,564, and 34,180 cd/m^2^ (Table 1).

### 3.3. Photoelectric Performance of Blue Exciplex BioLEDs

As one of the three primary colors, blue light is essential for panchromatic displays and illumination sources, therefore, blue light materials are very important in the OLED field. By adding PEDOT:PSS film to enhance the generation of electrons and holes in the EML, we have prepared dark blue exciplex doped-type BioLEDs with remarkable efficiency. Here, we are using organic material TCTA doping 1,3,5-tris(1-phenyl-1H-benzimidazol-2-yl) benzene (TPBi) as the main functional layer, TPBi as ETL.

**Figure 5:**
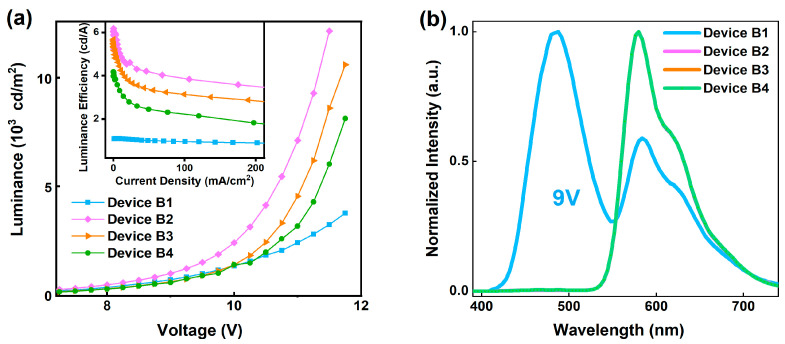
(**a**) L-V curve of devices A1–A4, inset show luminance efficiency-V curve of devices A1–A4, (**b**) EL spectra of at 9 V of devices A1–A4.

Similarly, the maximum luminance of device B1 (without PEDOT:PSS), B2 (PEDOT:PSS 1000 rpm), B3 (PEDOT:PSS 2000 rpm), and B4 (PEDOT:PSS 3000 rpm) are 3753, 12,150, 10,590, and 8111 cd/m^2^, respectively. The maximum luminance efficiency of devices B1–B4 are 1.1, 6.2, 5.7, and 4.1 cd/A, respectively (Figure 5a). By adding PEDOT:PSS HIL, the maximum luminance and luminance efficiency of blue exciplex BioLEDs increased by 224% and 464%. It proved that PEDOT:PSS as a HIL and buffer layer for exciplex BioLEDs can effectually improve the exciplex BioLEDs’ photoelectric performance.

The use of TCTA doped TPBi, on the one hand, and stable exciton recombination region can be formed in the EML to allow for more efficient charge recombination, resulting in a more stable spectrum of the Bio-device [42]. On the other hand, TCTA and TPBi were used as donor and acceptor materials, respectively, forming exciplex in the exciton recombination zones and emitting blue fluorescence [43]. The TCTA doped TPBi layer is deposited between the TCTA (hole transport layers) and TPBi (electron transport layer), facilitating the formation of an effective excited state. To demonstrate that TCTA and TPBi can effectively form exciplex, we measured the PL spectra of TCTA, TPBi, and TCTA:TPBi films with PL peaks of 384, 432, and 484 nm, respectively (Figure S2a). Meanwhile, the full width at half maximum (FWHM) of TCTA:TPBi film (87 nm) was broadened compared to the FWHM of TCTA and TPBi films (54 nm and 43 nm, respectively). The PL spectrum of TCTA:TPBi was significantly red-shifted and the FWHM of TCTA:TPBi was significantly larger than that of TCTA and TPBi, indicating that TCTA:TPBi form exciplex.

In addition, triphenylamine compounds containing triphenylamine electron-donating groups can not only form dimer to produce bimolecular luminescence (excimer or electromer), but also form heterogeneous bimolecular luminescence (exciplex, electroplex) with electron-accepting materials. Therefore, to study the bimolecular excited state luminescence of TCTA, we fabricated a monolayer organic layer device B: ITO/MoO_3_(10 nm)/TCTA(50 nm)/LiF(1 nm)/Al(120 nm). Figure S2b shows the EL spectra of device B and device B1 at 7 V. The EL spectrum of device B has two luminescence peaks at 432 and 584 nm, and the comparison with the PL spectrum of TCTA films shows that the luminescence peak at 432 nm is present in both PL and EL, while the luminescence peak at 584 nm is only observed in EL. This indicates that the luminescence peak at 432 nm is the monomer luminescence of TCTA and the EL peak at 584 nm is the electromer emission of the bimolecular excited state [44]. The EL spectrum of device B1 has three luminescence peaks at 488, 584, and 628 nm, the EL peak at 488 nm is the exciplex luminescence of TCTA:TPBi, the EL peak at 584 nm is the electromer emission of the bimolecular excited state, and the EL peak at 628 nm is the electroplex luminescence of TCTA:TPBi. In other words, under the action of the external electric field, electrons and holes are captured and cross-jumped by benzylamine groups in different TCTA molecules, resulting in the emission of electromer.

The EL spectra of devices B1–B4 were measured to verify the effect of PEDOT:PSS add on the luminance efficiency mechanism of the Bio-device. The EL peaks of device B1 are characteristic peaks of the exciplex and electromer (488 nm and 584 nm), while EL peaks of devices B2–B4 are characteristic peaks of the electromer and electroplex (584 nm and 628 nm) [45]. As mentioned previously, with the decrease of PEDOT:PSS spin-coated speeds, the concentration of holes increases. This leads to the movement of the electron–hole recombination region toward the EML/HTL interface as well as the decrease of the luminance intensity of the exciplex. As a result, the electron and hole composite probability in the EML increases and the luminance efficiency of the Bio-device is enhanced.

## 4. Conclusions

In this paper, the focus was on investigating the impact of PEDOT:PSS on the electroluminescence (EL) performance of doped-type BioLEDs using Alq_3_:Rubrene and TCTA:TPBi. Additionally, the study aimed to analyze the exciton recombination region within the Bio-device. By adding PEDOT:PSS films, the concentration of the hole in the EML is enhanced and the hole injection is improved. As a result, the PEDOT:PSS-based Alq_3_:Rubrene BioLEDs achieve higher luminance (44,010 cd/m^2^) and higher luminance efficiency (8.1 cd/A), which are increased by 186% and 478%, respectively, compared to that without PEDOT:PSS BioLEDs. Similarly, the maximum luminance of blue TCTA:TPBi exciplex-type BioLEDs increased by 224%, and the luminance efficiency increased by 464%. Significantly, one crucial finding in our study was that with the increasing thickness of the PEDOT:PSS layer, the exciton recombination region tends to shift towards the interface between the emitting layer (EML) and the hole transport layer (HTL). This phenomenon is noteworthy because it signifies that a thicker PEDOT:PSS layer helps to confine the excitons more effectively within the EML. By better confining excitons within the EML, the efficiency of the doped-type BioLEDs is effectively improved. In particular, as the thickness of PEDOT:PSS increases, the number of S_1,Rub_ and T_1,Rub_ increases, and the TTA process is enhanced, resulting in the enhanced luminescence of the Bio-devices. Therefore, the systematic study in this paper reveals not only the regulation of the concentrations of electrons and holes in the EML by using PEDOT:PSS as the HIL and hole buffer layer, but also the regulation of exciton recombination region in PEDOT:PSS-based doped-type BioLEDs by different PEDOT:PSS spin-coated speeds.

## Figures and Tables

**Table 1:** EL characteristics and MEL values of the devices A1–A4.

Device	MEL Values (%) ^1^	Luminance (cd/m^2^) ^1^	Maximum Luminance (cd/m^2^)
A1	−0.80	2885	15,400
A2	−1.73	8022	44,010
A3	−1.60	5959	39,564
A4	−1.36	4026	34,180

^1^ when the bias I is 400 μA.

## Data Availability

The datasets used and analyzed in the current study are available from the corresponding author on reasonable request.

## Supplementary Materials

The following supporting information can be downloaded at: https://www.mdpi.com/article/10.3390/polym15153275/s1, Figure S1: L-V curve of devices A1–A4; Figure S2: the photoluminescence (PL) spectra TCTA, TPBi, and TCTA:TPBi films and electroluminescence (EL) spectra of TCTA based Bio-device and TCTA:TPBi based Bio-device.
