# Supplementary material for: Effect of Conductive Polymers PEDOT:PSS on Exciton Recombination and Conversion in Doped-Type BioLEDs
Source: Polymers (Basel). 2023 Aug 2;15(15):3275. doi: 10.3390/polym15153275 (PMC10421517; doi:10.3390/polym15153275)
Supplement: Supplementary file 1 [file polymers-15-03275-s001.zip › polymers-2519529-supplementary.pdf]

Supporting Information

Effect of Conductive Polymers PEDOT:PSS on Exciton Recombination and Conversion in Doped-type BioLEDs

*Jiayi Song, Yunxia Guan *, Cheng Wang, Wanjiao Li, Xi Bao and Lianbin Niu **

College of Physics and Electronic Engineering, Chongqing Normal University,

Chongqing, 401331, China

Corresponding Author

*E-mail: (niulb03@126.com; utk_lili@126.com)

This file includes:

S1. OLEDs testing date.

L-V curveof devices A1-A4

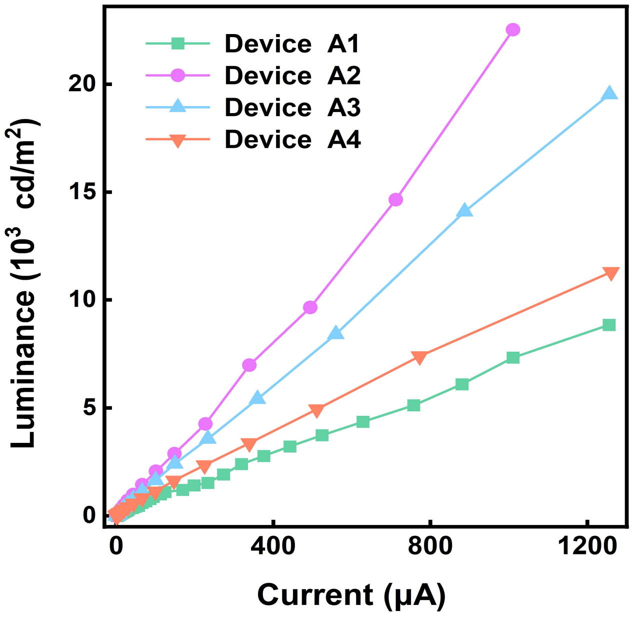

Figure S1 L-V curve of devices A1-A4 (device A1 without the PEDOT:PSS HIL, device A2 with the PEDOT:PSS HIL of 1000 rpm, device A3 with the PEDOT:PSS HIL of 2000 rpm, device A4 with the PEDOT:PSS HIL of 3000 rpm).

The photoluminescence (PL) spectra and electroluminescence (EL) spectra

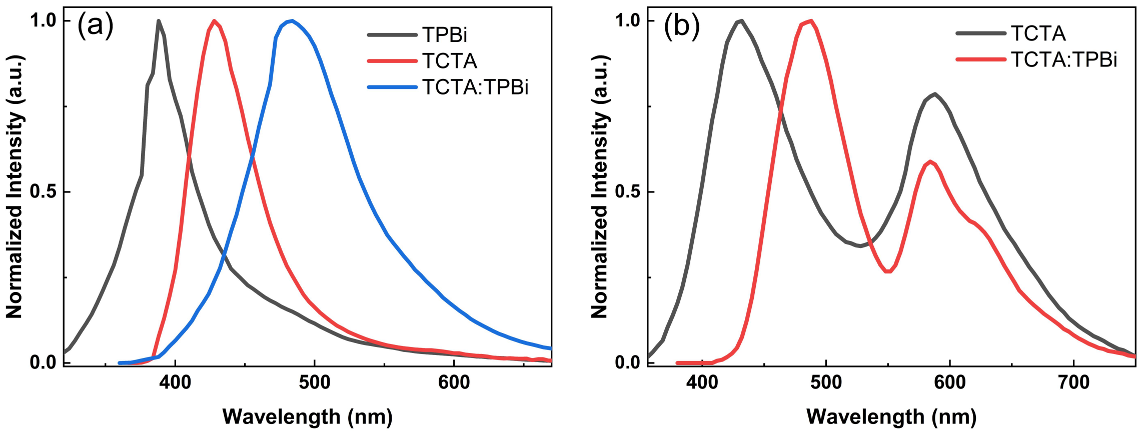

Figure S2 EL (a) PL spectra of TCTA, TPBi, and TCTA:TPBi films; (b) EL spectra of TCTA based device and TCTA:TPBi based device.
